# Comparison of Risk Factors Related to Intraventricular Hemorrhage between Preterm Infants Born After Normal and in Vitro Fertilization Conceptions

**Published:** 2019

**Authors:** Mamak SHARIAT, Parisa MOHAGHEGHI, Zahra FARAHANI, Nasrin KHALESI, Maryam NAKHOSTIN

**Affiliations:** 1 Maternal & Child Health Specialist, Maternal-Fetal & Neonatal Research Center, Tehran University of Medical Sciences, Imam Khomeini Hospital,Tehran, Iran.; 2 Neonatologist, Pediatrics Department, Ali-Asghar Hospital, Iran University of Medical Sciences, Tehran, Iran.; 3 Department of Infectious Diseases,Tehran University of Medical Sciences, Tehran, Iran

**Keywords:** Intraventricular hemorrhage, In vitro fertilization, Natural conception

## Abstract

**Objectives:**

We aimed to compare the level of significance of risk factors related Intraventricular hemorrhage (IVH) between preterm infants born after IVF and non-IVF conceptions.

**Materials & Methods:**

This historical cohort study was done in four Iranian Hospitals in 2013-2014. Overall, 155 preterm newborns were divided into case (IVF) and control (normal conception) groups. Both groups' demographic data were extracted and recorded. The incidence of IVH and its grades were compared between case and control groups. Significant related risk factors were also considered.

**Results:**

No differences were observed between 2 groups except for gestational age and mode of delivery. The incidence of IVH especially grades II and III were significantly higher in the case group (*P*=0.003). Results showed no correlations between Gestational age (GA), birth weight and number of gestations with the incidence of IVH in the case group (0.059, 0.85 and 0.49, respectively). On the other hand, among GA, birth weight and number of gestations; multi gestations (*P*=0.0001) was an effective risk factor for IVH occurrence in the controls.

**Conclusion:**

The incidence of IVH in the IVF group was significantly higher than in the non-IVF group. IVF as an independent risk factor may cause high-grade IVH; however, in the controls, multi gestational pregnancy (*P*=0.0001) was an effective risk factor for IVH occurrence.

## Introduction

Intraventricular hemorrhage (IVH) as an important cause of neonatal morbidity and mortality is frequently documented among premature infants (1). There are inverse correlations between the risk of IVH and neonate's gestational age or birth weight (2). The incidence of symptomatic IVH is 0.27–0.49 per 1000 live births (1). This rate among extremely low birth weight neonates was reported higher than 27% (3). 

Depending on types of IVH (epidural, subdural, subarachnoid, intraventricular and intraparenchymal) neurological sequels and developmental disorders are common (4, 5). Although both low birth weight and gestational age are the major risk factors, other maternal and neonatal conditions are involved; smoking, breech presentation, gender, premature rupture of membranes, intrauterine and post-natal infections, mode of delivery, prolonged labor, hypercapnia or rapid bicarbonate infusion, cerebral blood flow disturbance, hypotension, metabolic, hematologic and respiratory complications (2, 6-8).

Ten percent of couples suffer from infertility and are introduced for in vitro fertilization (IVF) as an effective treatment (9). Recent studies have shown an association between IVH and IVF treatment; assisted reproductive techniques, particularly IVF, are significant risk factors for IVH (OR: 4.34; 95% CI: 1.42–13.3). A higher incidence rate of IVH was indicated in 73 neonates from IVF pregnancies in compare to 148 subjects from normal conceptions. Overall, 517 preterm babies born after IVF and natural conception, preterm mortality and morbidity did not differ between the 2 groups, except for IVH (2,10-12).

Iran as one of the pioneers of ART (assisted reproductive techniques) in Middle East from 1980, now has more than hundreds of IVF cycles a year (13). Many studies have investigated the effects of IVF pregnancies on neonatal mortality and morbidity recently (14-16). Some studies also have determined risk factors for Intraventricular Hemorrhage in Infants born after ART (10-12). 

We aimed to compare the level of significance of risk factors related IVH between preterm infants born after IVF and non-IVF conceptions. 

## Material &Methods

In this historical cohort study, newborns with gestational age<34 wk born and hospitalized at NICU in 4 Iranian Hospitals (Ali-Asghar, Akbar Abadi, Rasoul Akram and Vali-Asr Hospitals, Tehran, Iran) were enrolled during 2013 and 2014. According to method of conception, all participants' medical records were collected and studied. Based on type of conception, 155 preterm newborns were divided into case (IVF) and control (non IVF) groups. Both groups' demographic data including birth weight, gestational age (based on mother's Last Menstrual Period (LMP) and ultrasound measures), gender, duration of NICU hospitalization, mode of delivery, brain imaging results related IVH and its grade were extracted and recorded. 

We excluded newborns due to lack of data, receiving prophylactic medication like indomethacin, death before hospital discharge, chorioamnionitis cases and urgent cesarean sections. The incidence of IVH and its grades were compared between case and control groups. Significant related risk factors were also considered.

The study was approved by the Institutional Review Board of Tehran University of Medical Sciences according to Helsinki declaration in 2013. Our gathered data were confidential and no extra cost was constrained on our participants. Informed consent was taken from parents before the study.

Statistical analysis was done with SPSS ver.18 software (Chicago, IL, USA). Frequency and mean± standard deviation were considered for analysis of quantitative and qualitative variables. Chi-square and *t*-test were applied for comparison of demographic variables and outcome of 2 groups. Significance level was considered 95% (*P*<0.05). The comparison of effective variables on the IVH incidence in each group was performed by binary logistic regression and ANOVA tests. With the proposed sample size of 155, the study had a power of 75% and an alpha error of 0.05.

## Results

There were 93 and 62 preterm newborns in the control and case groups, respectively. With regard to demographic characteristics, no differences were observed between 2 groups except for gestational age and mode of delivery; mean gestational age in the control group was significantly lower than in the case group (30.19 vs. 31.48 wk, *P*=0.001) (Table 1). The mode of all deliveries in the case group and in 84% of the control group was C/S section (*P*=0.001). 

**Table 1:** Demographic characteristics of two groups*

Variable	Case group n=62	Control group n=93	*P* value
Mean birth weight (gr)	1553.87± 429.8	1551.25± 508.3	0.97
Female gender	51.6 %	46%	0.512
Mean days of hospitalization	21.38 ± 13.9	18.9 ± 20.4	0.41
Mean G/ Age** (weeks)	31.48± 2.27	30.19± 2.31	0.001
C/S	62 (100%)	78 (84%)	0.001
Sex			
Male female	30 (48%) 32 (52%)	56 (60.6%) 37 (39.4%)	0.2

* Chi-square and t-test

** G/ Age; gestational age

**Table 2:** **Comparison of variables between case and control groups** *

Variable	Case group n=62	Control group n=93	*P* value
Multi gestations			
Twin pregnancy Triplet pregnancy	28 (45) 18 (29)	12 (13) 2 (3)	0.0001
IVH			
1^st^ grade 2^nd^ grade 3^rd^ grade	21 (34) 8 (38) 8 (38) 5 (23)	12 (13) 9 (75) 2 (17) 1 (8)	0.003

* Chi square test

**Table 3:** Comparison of risk factors of IVH in the case and control groups*

Variable	Case group *P****-***value	Control group *P***-**value
G/Age**	0.059	0.15
Birth weight	0.85	0.26
Multi gestations	0.49	0.0001

* Chi-square and t-test,

** G/ Age; gestational age

**Table 4:** Risk factors of IVH*

		B	S.E.	Sig.	OR	95% CI. for EXP(B)	
						Lower	Upper
	Group	4.302	1.219	.000	73.880	6.773	805.844
	GA	.606	.207	.003	1.834	1.222	2.751
	Multiple pregnancy	3.640	.906	.000	38.078	6.449	224.830

* Binary logistic regression and ANOVA tests

The incidence of IVH especially grades II and III were significantly higher in the case group (*P*=0.003). Grade IV IVH was not seen in either group. The number of twin and triplet pregnancies were significantly higher in the case group than in another group (*P*=0.0001). Comparison of variables between 2 groups is demonstrated in Table 2. Regarding to find correlations between the incidence of IVH and some risk factors like GA, birth weight and number of gestations, statistical analysis was also performed within each group. Results showed no correlations between GA, birth weight and number of gestations with the incidence of IVH in the case group (*P*=0.059, *P*=0.85, and *P*=0.49, respectively). On the other hand, among above variables, multi gestational pregnancy was an effective risk factor (*P*=0.0001) for IVH occurrence in the controls (Table 3). 

To omit confounding factors and interactions between variables, binary logistic regression was applied. IVH was considered as dependent variable while gestational age, prenatal complications, multi gestations, type of delivery, sepsis, resuscitation requirement, sex, birth weight, and IVF were considered as independent factors. Each independent factor including gestational age, multiple gestation and IVF affect positively on the prevalence of IVH. Among these factors, IVF had strongest effect; IVF with OR 73, multi gestations with OR 38 and low gestational age with OR 1.8 independently increased the risk of IVH (Table 4).

## Discussion

Result of the present study showed that the incidence of IVH especially grade II and III in the IVF group was significantly higher than in the counterpart group (51% vs. 25%). Inconsistent with our result, other studies pointed to a high frequency of neonatal complications including intraventricular hemorrhage among infants born after artificial fertilization. Manoura showed IVF pregnancy as a significant risk factor for IVH (11). The incidence of IVH among preterm neonates in the IVF group was significantly higher than those in the non-IVF group (*P*<0.001) (12). On the other hand, a cohort study showed no significant difference with regard to the IVH incidence among 90 very premature infants in 2 groups; born after IVF or normal conception (17).

Mean gestational age in the control group was significantly lower than in the case group. In contrast to our results, of 940 term and preterm newborns, gestational age in the IVF group was significantly lower than in the non-IVF group (31 vs. 32, *P*<0.001) (12). IVH occurrence was independent of gestational age (GA) and is frequently happened within the first 24-48 h after birth (18). Regarding birth weight, no difference was observed between 2 groups. This finding was confirmed by another study (19).

Our main finding revealed that IVF as an independent risk factor could develop high-grade IVH. We could not find any associations between the incidence of IVH and birth weight, gestational age and multiple gestations in the IVF group. In accordance with our results, in vitro fertilization may be a merely risk factor for IVH. In this regard, maternal complications related to infertility like using medication, vasoreactivity or platelet aggregation status may involve in the increasing the risk of neonatal IVH (20). Several maternal factors were pointed such as maternal chorioamnionitis, infection, inflammation and antenatal maternal hemorrhage that possess predictive value for the IVH development in neonates (18). However, other studies have shown an inverse correlation between IVH and birth weight (2-3). Ballabh indicated a high incidence of IVH (up to 40%-50%) in VLBW and extremely premature infants (21). Our participants in both groups were not very (<1500 gr) or extremely low birth weight (<500-750 gr) infants. Mean birth weight of all subjects were >1500 gr. Moreover, although multiple pregnancies was an effective risk factor for IVH occurrence in our control group, in the IVF group this correlation was not observed. Compatible to this finding, multiple gestation can increase the morbidity rate in premature neonates born after naturally conceived unlike in neonates with IVF conception (12).

Unlike in the case group, we observed that multi gestation pregnancy was an effective risk factor for IVH occurrence in the controls. Incompatible with our result, multiple gestations were a significant risk factor for IVH among white preterm infants (*P*<0.05) (21). On the other hand, no correlation was found between intracranial hemorrhage (grade>3) and cystic periventricular leukomalacia and multiple gestations (22).

A limitation of our study was few numbers of the IVF cases in our hospitals and a long time requirement for data gathering. However, we tried to solve this problem with retrograde method (historical cohort study) and multi-centered data gathering in more than one hospital. 

Moreover we did not consider some other risk factors like birth length, respiratory distress syndrome, mechanical ventilation, antenatal steroids administration and Apgar score that certainly could provide more informative data.

**In conclusion, **the incidence of IVH in IVF group was significantly higher than in non-IVF group. IVF conception itself is a risk factor for incidence for IVH and its higher grades. Multi gestation was a risk factor for IVH incidence in natural pregnancies.
